# A Multidimensional Hyperjerk Oscillator: Dynamics Analysis, Analogue and Embedded Systems Implementation, and Its Application as a Cryptosystem

**DOI:** 10.3390/s20010083

**Published:** 2019-12-21

**Authors:** Tsafack Nestor, Nkapkop Jean De Dieu, Kengne Jacques, Effa Joseph Yves, Abdullah M. Iliyasu, Ahmed A. Abd El-Latif

**Affiliations:** 1 Research Unit of Laboratory of Automation and Applied Computer, Electrical Engineering Department of IUT-FV, University of Dschang, P.O. Box 134 Bandjoun, Cameroon; 2 Research Unit of Laboratory of Condensed Matter, Electronics and Signal Processing, Department of Physics, Faculty of Sciences, University of Dschang, P.O. Box 67 Dschang, Cameroon; 3 Department of Electrical Engineering and Industrial Computing, University Institute of Technology, P.O. Box 8698 Douala, Cameroon; 4 Department of Communications, Technical University of Cluj-Napoca, 26-28 Baritiu Street, 400027 Cluj-Napoca, Romania; 5 Electrical Engineering Department, College of Engineering, Prince Sattam Bin Abdulaziz University, Al-Kharj 11942, Saudi Arabia; 6 School of Computing, Tokyo Institute of Technology, Yokohama 226-8502, Japan; 7 School of Computer Science and Technology, Changchun University of Science and Technology, Changchun 130022, China; 8 Mathematics and Computer Science Department, Faculty of Science, Menoufia University, P.O. Box 32511 Shebin El-Koom, Egypt; 9 Department of Cybersecurity, College of Computer Science and Engineering, University of Jeddah, P.O. Box 23890 Jeddah, Saudi Arabia; 10 School of Computer Science and Technology, Harbin Institute of Technology, Harbin P.O. Box 150080, China

**Keywords:** hyperjerk oscillator, embedded systems, multiple coexisting attractors, information security, image encryption

## Abstract

A lightweight image encryption algorithm is presented based on chaos induction via a 5-dimensional hyperjerk oscillator (5DHO) network. First, the dynamics of our 5DHO network is investigated and shown to exhibit up to five coexisting hidden attractors in the state space that depend exclusively on the system’s initial values. Further, a simple implementation of the circuit was used to validate its ability to exhibit chaotic dynamical properties. Second, an Arduino UNO platform is used to confirm the usability of our oscillator in embedded system implementation. Finally, an efficient image encryption application is executed using the proposed chaotic networks based on the use of permutation-substitution sequences. The superior qualities of the proposed strategy are traced to the dynamic set of keys used in the substitution process which heralds the generation of the final ciphered image. Based on the average results obtained from the entropy analysis (7.9976), NPCR values (99.62), UACI tests (33.69) and encryption execution time for 512 × 512 images (0.1141 s), the proposed algorithm is adjudged to be fast and robust to differential and statistical attacks relative to similar approaches.

## 1. Introduction

A lot of development in internet and multimedia technology has been witnessed over the past decade. This has facilitated seamless exchange and transfer of confidential information. Moreover, the confidentiality, authentication and integrity (CIA) triad is widely cited as the cornerstone of information security. Among others, it provides the effective copyright protection and confidentiality needed in business, entertainment, healthcare, military, etc. communication. Encryption of sensitive data is one of the most important information security strategies that is necessary for confidentiality. Available encryption and decryption algorithms used to forestall malicious attacks from unauthorised parties include DES, 3-DES, AES, IDEA, RSA, etc. [1,2,3]. However, due to data capacity resource demands and high correlation among pixels in image files, these standard algorithms withered in providing efficient protection for images [4]. Moreover, although widely used in cryptanalysis, computer science and electrical engineering, pseudo random number generators (PRNG) are less effective in cryptography. As a solution, chaos-based protocols have continued to gain traction in mitigating image security issues [5,6,7,8,9,10,11].

Many studies have focused on ergodicity, deterministic dynamics, unpredictable behaviors, non-linear transformation, sensitivity dependence, etc. of the system. Research efforts have explored the use of striking periodic attractors, chaotic attractors or hyperchaotic attractors, antimonotonicity, period doubling, hysteresis, coexisting bifurcations, etc. in investigating the dynamic behaviours of systems and their possible applications [12,13,14,15,16,17]. Interestingly, some of these characteristics have been found useful in image encryption [6,18]. In [19], Shuqin and collaborators presented a novel encryption algorithm based on chaos and SHA-256 whose experimental results show that it was efficient and reliable. This was further enhanced in [12], wherein Quing et al. proposed an S-box design algorithm based on a new compound chaotic system. In their effort, in [20], Biham et al. demonstrated the exploitation of the weakness inherent to piecewise linearity of the tent map and its limitation to 75 random bits to violate the intensity of the system using a pair of known and chosen plain text attacks. Similarly, in [21], Baptista suggested the use of a chaotic attractor, plaintext and logistic map for image encryption. 

Due largely to its relatively cheap pricing and utility, a large community has arisen around the Arduino open-source computer hardware and software platforms and through them hundreds of free scripts for different projects are easily available. Mauricio et al., presented a communication system based on chaotic logistic maps and an experimental realization of it using Arduino board. Therein, the input message was moderated using a Delta modulator and encrypted using a logistic map. The key signal is also encrypted using the same logistic map but with different initial conditions. On the receiver side, the binary-coded message is decrypted using the encrypted key signal that is sent through a communication channel. In [22], Adolfo and collaborators designed, implemented and evaluated a compact two axes solar tracking system. The system incorporates a video processing-based sensor connected to an Arduino board that computes a sun-positioning algorithm. The main advantage of such systems is the elimination of expensive computing systems where closed loop solar tracking is facilitated via simple, low cost networks with minimal configuration. The assessment of their results indicates the system’s efficiency relative to some existing approaches. Arduino have also been used to handle sensor- and vision-based image processing techniques [23,24]. In [24], Nikhil et al. studied a traffic monitoring system for road vehicle traffic. The study in [25] is aimed at minimising human support and avoiding accidents on the roads. 

Motivated by the applications of Arduino networks highlighted above, we consider its deployment in the embedded system implementation [26] of our 5DHO chaos generation network. The contributions of our study are enunciated in the sequel. 

### Our Contributions

In this study, we present a multidimensional oscillator (5DHO) network for use as a chaos generator and cubic nonlinearity to the network in [27]. The choice of the proposed system utilises semiconductor diodes rather than analogue multipliers. Specifically, we utilise a network of diode operational amplifiers and resistors to derive a piecewise linear (PWL) approximation of the cubic and quadratic functions needed for chaotic non-linearity [28]. Therefore, low cost, convenient circuits whose output is the square or cube of their input are used to realise 5-D hyperjerk characteristics. Additionally, an Arduino UNO board is used to establish the dynamics of our oscillator and its usability in embedded systems technologies.

Finally, a lightweight encryption algorithm is designed based on permutation-substitution boxes and the sequences of the 5DHO. Our strategy offers a dynamic set of keys for use in generating the ciphered image.

The remainder of paper is structured as follows: Section 2 introduces the dynamics of the proposed multidimensional hyperjerk oscillator. Analogue and embedded systems implementations of the proposed network are presented in Section 3. Following that, Section 4 presents our proposed encryption and decryption procedures as well as their performance analysis are also reported. 

## 2. Dynamics of the Proposed Multidimensional Hyperjerk Oscillator

### 2.1. Mathematical Formulation of Proposed 5-D Hyperjerk Oscillator Network

The mathematical model of the proposed 5-D hyperjerk system is formalised in the set of differential equations in (1).

(1) $$\left\{ \begin{array}{l} {\dot{x}}_{1}=x_{2}\\ {\dot{x}}_{2}=x_{3}\\ {\dot{x}}_{3}=x_{4}\\ {\dot{x}}_{4}=bx_{5}\\ {\dot{x}}_{5}=-a_{0}x_{5}-a_{1}x_{3}-a_{2}x_{2}-a_{3}x_{1}-y \end{array}\right.$$

where $y=a_{4}x_{4}(x_{4}-l_{1})(x_{4}-l_{2})=a_{4}l_{1}l_{2}x_{4}-a_{4}(l_{1}+l_{2})x_{4}^{2}+a_{4}x_{4}^{3}$ is the nonlinear function containing both cubic and quadratic nonlinearities. In the present study, these nonlinearities are implemented without any analog multiplier. $x_{i}(i=1,2,3,4,5)$ are state variables and and $a_{0}\in \left[0.8;2\right],$$a_{1}\in \left[2.7;6\right],$ $a_{2}\in \left[1;5\right],$ $a_{3}\in \left[0.1;1.5\right],$$a_{4}\in \left[0.1;1.5\right],$b∈[0.1;1.5],$l_{1}\in \left[0;3\right],$$l_{2}\in \left[0;3\right]$ are positively valued constants. 

The fourth order Runge-Kutta algorithm with a trifling integration step to will be used analyse the behaviour of the 5-D hyperjerk system in (1) through Hopf bifurcation diagrams, Lyapunov exponents and phase space trajectories.

### 2.2. Fixed Point and Stability

Since the unique equilibrium (origin) point O(0,0,0,0,0) of the proposed model in (1) is the solution of the nonlinear system: ${\dot{x}}_{1}={\dot{x}}_{2}={\dot{x}}_{3}={\dot{x}}_{4}={\dot{x}}_{5}=0$, the stability of the equilibrium can be described by the following characteristic equation:

(2) $$\lambda^{5}+a_{0}\lambda^{4}+a_{4}bl_{1}l_{2}\lambda^{3}+a_{1}b\lambda^{2}+a_{2}b\lambda +a_{3}b=0$$

As the real parts of the correlated eigen values are always negatively valued; the equilibrium is stable for the entire region of system parameters. For instance, if we set ${b=3;\;a}_{0}={1.5;\;a}_{1}{=3;\;a}_{2}{=2;\;a}_{3}{=1;\;a}_{4}=1;\;l_{1}=1;\;l_{2}=2.6$ then its eigen values can be calculated as:

(3) $$\begin{array}{l} \lambda_{1}=\;-\;0.1194\;+\;2.5677i;\;\lambda_{2}=\;-\;0.1194\;-\;2.5677i;\;\lambda_{3}=\;-\;0.8425\;+\;0.0000i\\ \lambda_{4}=\;-\;0.2094\;+\;0.7036i;\;\lambda_{5}=\;-\;0.2094\;-\;0.7036i \end{array}$$

It is a general conclusion that since the equilibrium point is always stable, it can be predicted that a point attractor coexists with a strange attractor [27,28]. This is further clarified later in the next section.

### 2.3. Bifurcations and Multistability

**Figure 1:**
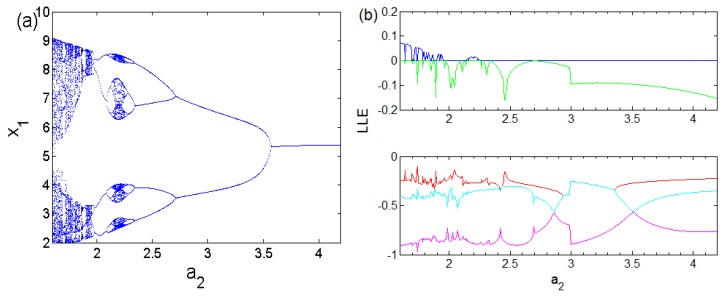
Dynamics of the 5-D oscillator for conditions ${(a}_{0}\;,\;a_{1}\;,\;a_{3}\;,\;a_{4}\;,\;b\;,\;l_{1}\;,\;l_{2})=(1.5\;,\;2.6\;,\;1\;,0.6,\;3\;,\;1\;,\;2.6)$. (**a**) bifurcation diagram and (**b**) Lyapunov spectrum.

**Figure 2:**
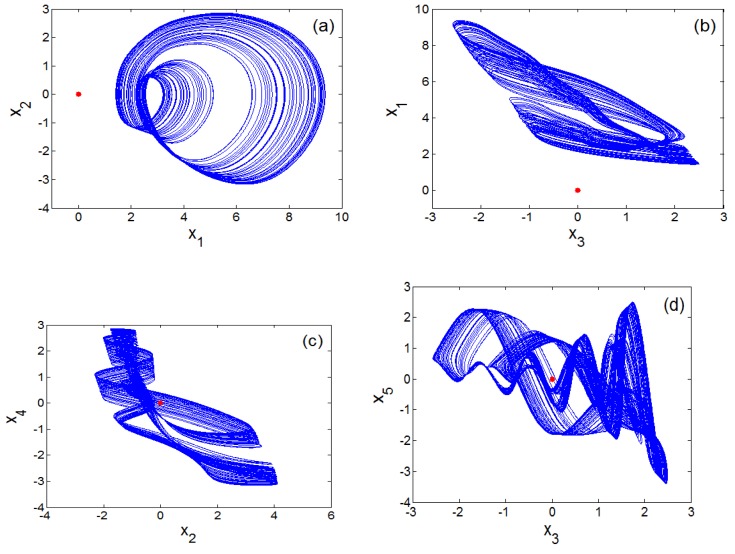
Four views of the 5D Hyperjerk attractor with stable equilibrium point (shown as a red dot) with various projections: (**a**) $x_{1}-x_{2}$, (**b**) $x_{3}-x_{1}$, (**c**) $x_{2}-x_{4}$, (**d**) $x_{3}-x_{5}$.

The bifurcations of an oscillator with respect to parameter *a*_2_ can be investigated when parameters values are assigned as ${(a}_{0}\;,a_{1}\;,a_{3}\;,a_{4}\;,\;b\;,\;l_{1}\;,\;l_{2})=(1.5\;,\;2.6\;,\;1\;,0.6,\;3\;,\;1\;,\;2.6)$. Figure 1a shows that the oscillator studied in our study exhibits the reverse period doubling paths to chaos with the primary value (6,0,0,0,0). It is patent that the oscillator experiences antimonotonicity behaviour. Lyapunov spectrum [29,30,31,32] is also used to attest the chaotic dynamics of the system (Figure 1b). Figure 2 provides four views of the 5-D Hyperjerk chaotic attractor where the stable equilibrium point is shown as a red dot.

**Figure 3:**
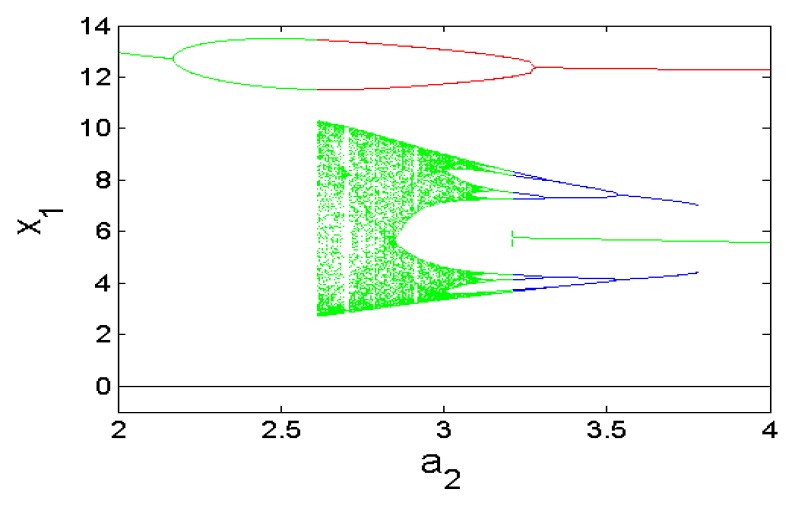
Dynamics of the 5-D Hyperjerk oscillator illustrating multistability.

**Figure 4:**
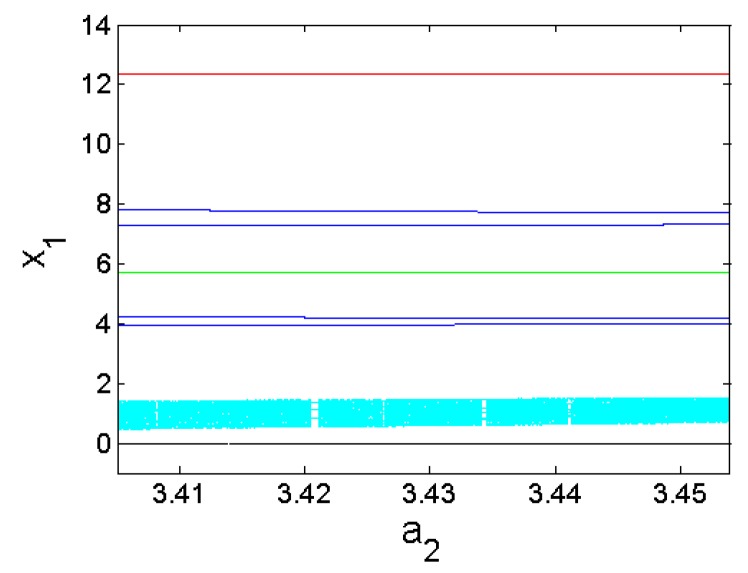
Blow-up of bifurcation plot in Figure 3 for the range $3.405\leq a_{2}\leq 3.455$.

Furthermore, coexisting bifurcation diagrams are used to illustrate the phenomenon of multistability in the system (1) (see Figure 3 and Figure 4) where we point out that these graphs are the plots of the local maximums of the variable x_1_ against parameter a_2_.

**Figure 5:**
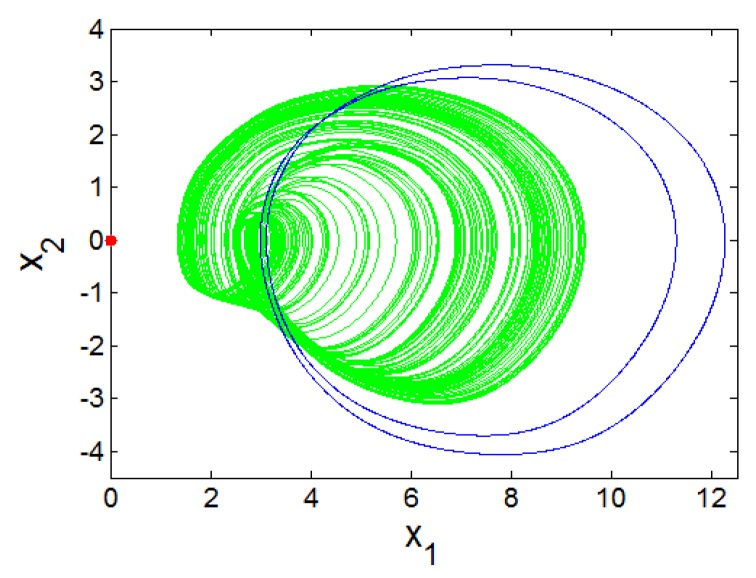
Evidence of strange attractor coexisting with period-2 limit cycle.

**Figure 6:**
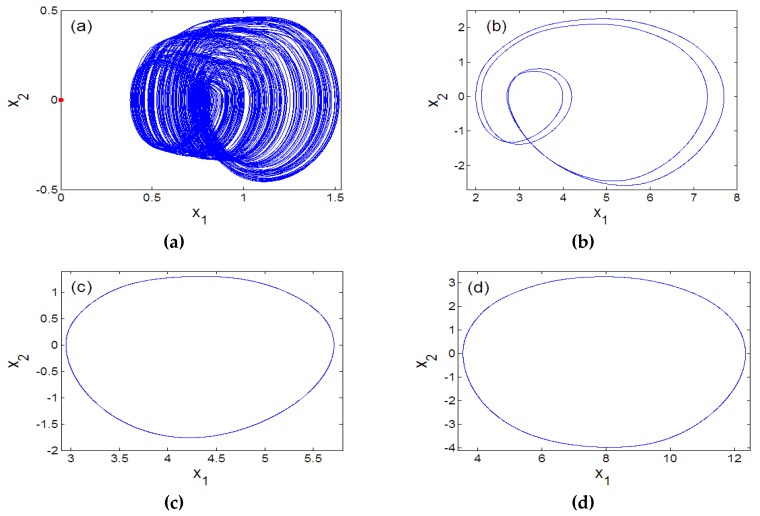
Illustration of coexistence of strange atrractor with limit cycles for different initial conditions: (**a**) (0.4,0,0,0,0) and (1.2,0,0,0,0); (**b**) (5.2,0,0,0,0); (**c**) (4,0,0,0,0); (**d**) (4.4,0,0,0,0).

While details of strategies used are presented in Table 1, it is evident from these graphics that up to four attractors can coexist (Figure 5 and Figure 6). Table 2 provides initial solution and system parameters in each case. Offset boosting is another striking behaviour observed in the system presented in (1). For illustration, (1) is rewritten by replacing the state *x*_1_ with *x*_1_+*k* as presented in (4).

(4) $$\left\{ \begin{array}{l} {\dot{x}}_{1}=x_{2}\\ {\dot{x}}_{2}=x_{3}\\ {\dot{x}}_{3}=x_{4}\\ {\dot{x}}_{4}=bx_{5}\\ {\dot{x}}_{5}=-a_{0}x_{5}-a_{1}x_{3}-a_{2}x_{2}-a_{3}(x_{1}+k)-a_{4}x_{4}(x_{4}-l_{1})(x_{4}-l_{2}) \end{array}\right.$$

**Figure 7:**
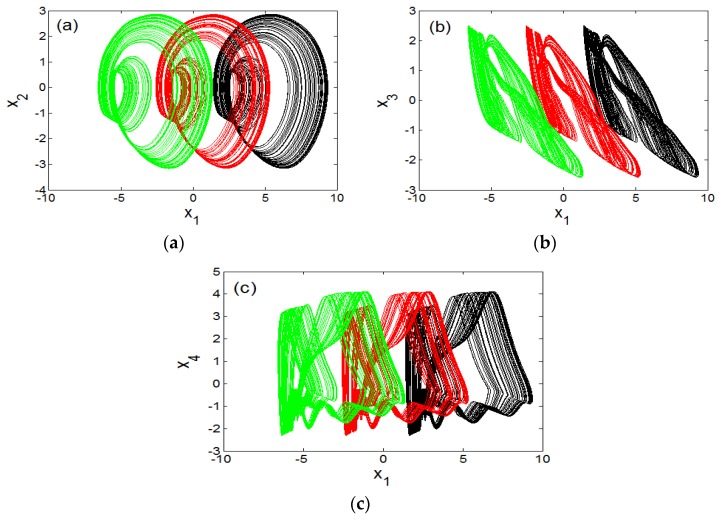
Offset-boosting of chaotic attractor for varying control parameter (*k*) values: (**a**) in $x_{1}\;-\;x_{2}$ plane, (**b**) in $x_{1}\;-\;x_{3}$ plane and (**c**) in $x_{1}\;-\;x_{4}$ plane for *k* = 0 (black) k = 4 (red), and k = 8 (green).

When switching parameter *k*, the chaotic signal *x*_1_ can be transferred from a bipolar signal to a unipolar signal as illustrated in Figure 7.

## 3. Experimental Analysis of Proposed Oscillator

### 3.1. Analogue Simulation Results on the Designed Circuit Using Spice 

**Figure 8:**
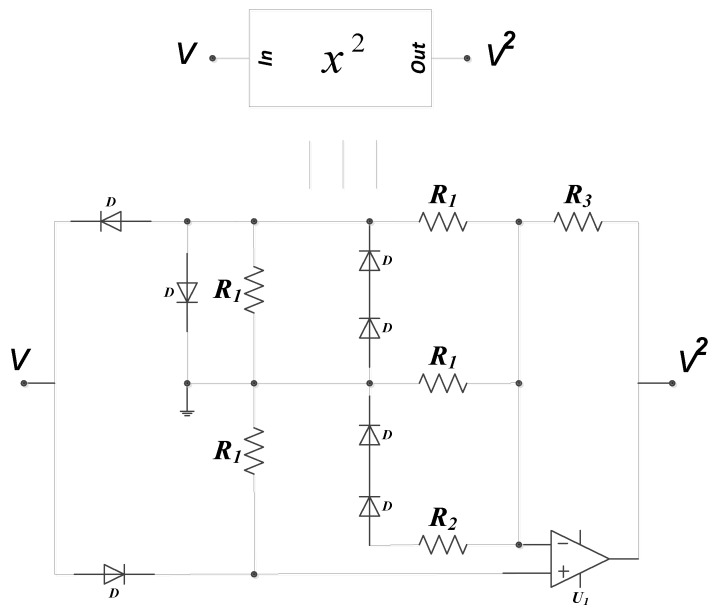
Design of square nonlinearity for circuit values $R_{1}=10k\Omega ;\;\;R_{2}=4k\Omega ;\;\;R_{3}=30k\Omega$.

**Figure 9:**
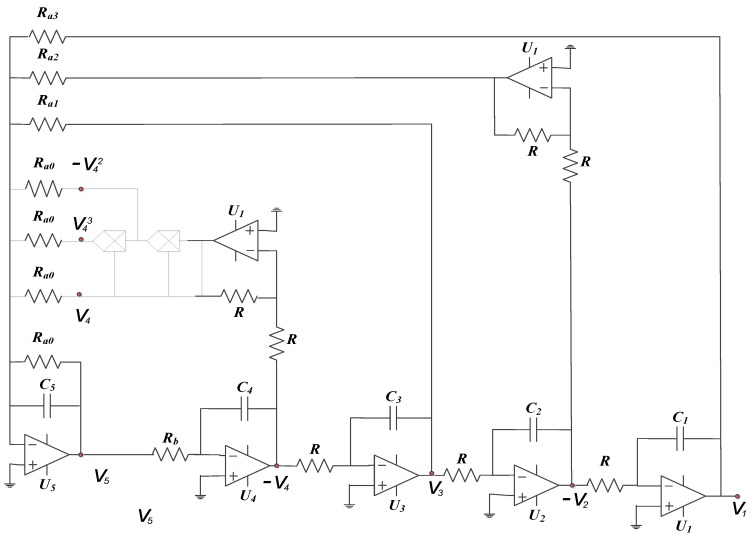
Circuit design for implementation of proposed 5-D hyperjerk system.

**Figure 10:**
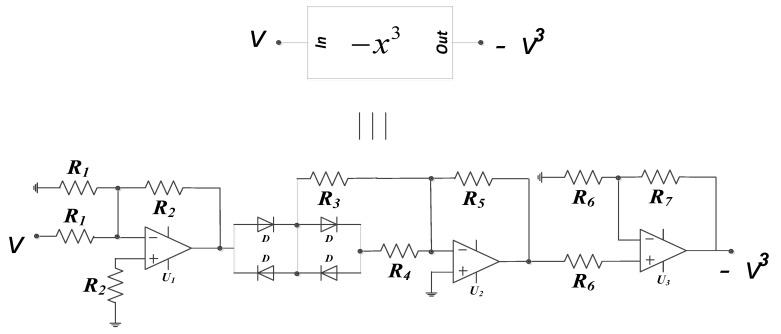
Design of the cube function for circuit values $R_{1}=200k\Omega ;$ $R_{2}=100k\Omega ;\;\;$ $R_{3}=12k\Omega ;$ $R_{4}=2k\Omega ;$ $R_{5}=15k\Omega ;\;\;R_{6}=10k\Omega$.

This section highlights the intricacies in the design of our proposed 5-D hyperjerk oscillator and its simulation. We start by recalling that this system exhibits both cubic and quadratic polynomials. The circuit in Figure 8 produces an output that is the square of its input, while the one in Figure 9 implements a PWL approximation of a circuit whose output is the cube of its input. These circuits are convenient for low-cost analogue realization of our proposed network that is depicted in Figure 10. Here, state variables *x_i_* (*i* = 1…5) of the system in (1) are associated with the voltages *v_i_* (*i* = 1…5) across the capacitors *C_i_* (*i* = 1…5) respectively. By linking the state variable *x_i_* (*i* = 1…5) with the voltages *v_i_* (*i* = 1…5) across the capacitors *C_i_* (*i* = 1…5), we derive circuit equations in the form presented in (5).

(5) $$\left\{ \begin{array}{l} C_{1}\frac{d\upsilon_{1}}{dt}=\frac{\upsilon_{2}}{R}\\ C_{2}\frac{d\upsilon_{2}}{dt}=\frac{\upsilon_{3}}{R}\\ C_{3}\frac{d\upsilon_{3}}{dt}=\frac{\upsilon_{4}}{R}\\ C_{4}\frac{d\upsilon_{4}}{dt}=\frac{\upsilon_{5}}{R_{b}}\\ C_{5}\frac{d\upsilon_{4}}{dt}=-\frac{\upsilon_{5}}{R_{a0}}-\frac{\upsilon_{3}}{R_{a1}}-\frac{\upsilon_{2}}{R_{a2}}-\frac{\upsilon_{1}}{R_{a3}}-\frac{\upsilon_{4}}{R_{1}}-\frac{\upsilon_{4}^{2}}{R_{2}}+\frac{\upsilon_{4}^{3}}{R_{3}} \end{array}\right.$$

Similarly, by rescaling time and other variables: $t_{e}=tRC;\;\;\upsilon_{i}=x_{i}nV_{T}\;(i=1,2,3)$ and subject to adjustments in parameter values in (6), the system in (5) can be seen to be identical to the one in (1).

(6) $$a_{0}=\frac{R}{R_{a_{0}}};\;\;a_{1}=\frac{R}{R_{a_{1}}};\;\;a_{2}=\frac{R}{R_{a_{2}}};\;\;a_{3}=\frac{R}{R_{a_{3}}};\;\;a_{4}l_{1}l_{2}=\frac{R}{R_{1}};\;\;a_{4}(l_{1}+l_{2})=\frac{R}{R_{2}};\;\;a_{4}=\frac{R}{R_{3}};\;$$

**Figure 11:**
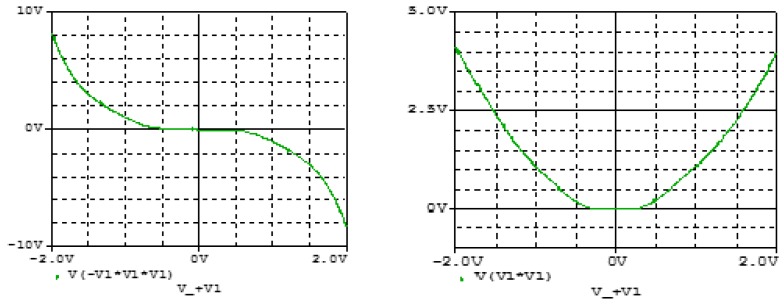
Transfer functions for the cube square functions as obtained Pspice simulation of the circuits in Figure 8 and Figure 10.

**Figure 12:**
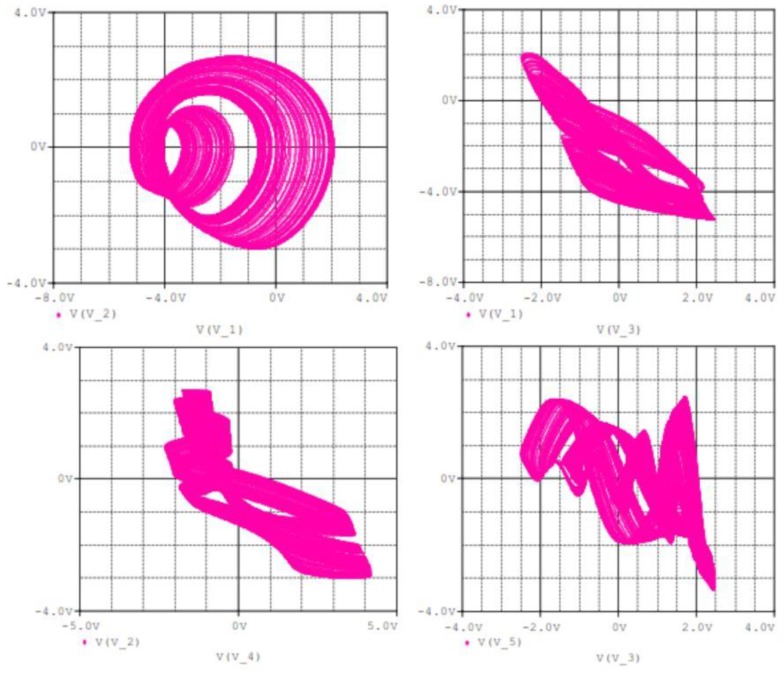
Phase plots for of the proposed 5D Hyperjerk system as observed via Pspice simulation of the network for component values listed in Table 3 and initial conditions set at (1, 1, 1, 1, 1).

**Figure 13:**
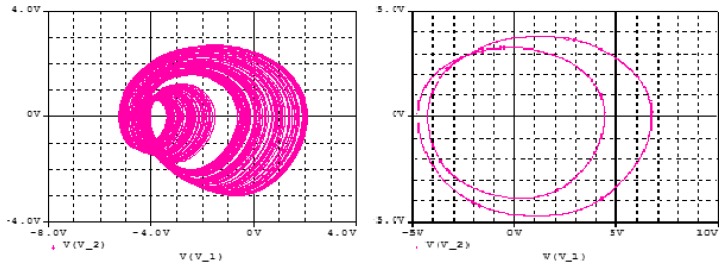
Evidence of presence of coexistence of hidden chaotic attractor with a hidden limit cycle as observed via Pspice simulation of the network for component values listed in Table 3 and initial conditions are set at (1, 1, 1, 1, 1) and (10, 0, 0, 0, 0).

Pspice simulation is used to validate the theoretical expectations of the circuit in Figure 9 in terms of coexistence of hidden attractors. $R_{a2}$ is used as the main control resistor and the rest of circuit components are fixed as mentioned in Table 3. The synergy between the theoretical results (i.e., in Figure 2 and Figure 5) and the Pspice simulation results (in Figure 11 and Figure 12) shows the feasibility of the suggested chaotic system with hidden attractors based on the stated electronic components whose evidence of presence of coexisting hidden chaotic attractors are presented in Figure 13.

### 3.2. Arduino Based Implementation of Proposed Oscillator

**Figure 14:**
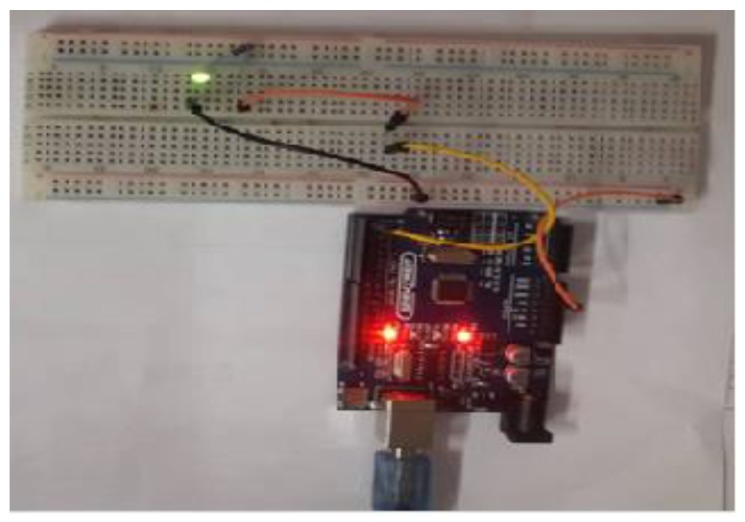
Chaos generation via experimental operation using Arduino Uno board.

While Field Programmable Gate Arrays (FPGA) are the popular option for providing configurable circuits practical implementation of embedded systems using chaos [33], recently some microcontroller-based chaotic systems have been considered due to their equally flexible and cheap pricing for different programming applications [34,35,36,37,38,39,40,41]. In this study, we use an Arduino UNO board platform to compute and visualise the solutions (for example, using an oscilloscope) of our chaos generator. The Arduino board used in our study is presented in Figure 14 and further details pertaining to its implementation are outlined in the sequel.

**Step 1**: Set pins 1 and 2 as outputs. The solutions of our chaotic oscillator will be written here.
**Step 2**: Define the discrete chaotic oscillator, its parameters and initial conditions under an infinite loop.
**Step 3**: Write the solutions of the discrete chaotic oscillator on Arduino pins. Pin 1 is activated when x2 > 0.5 and pin 2 is activated when x1 > 1.

**Figure 15:**
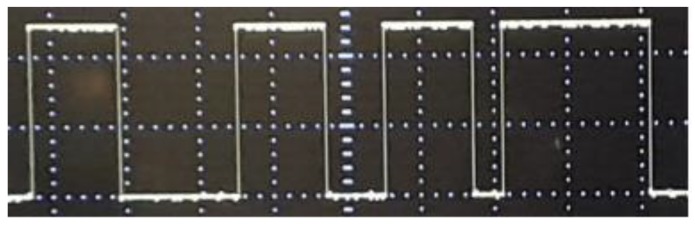
Experimental result at pin 5 of Arduino Uno.

The above algorithm is executed using the open-source platform Arduino 1.8.9 and the experimental result (in Figure 15) is recovered via traces on an oscilloscope connected at pin 2 with scales set at X = 2 V/div and Y = 500 ms/div.

Meanwhile, by using “*millis()*”function on the Arduino platform, the experimental time can be printed. Here, a readout of 0.064 ms for a 16 MHz crystal oscillator (the frequency of the quartz mounted on the card) using only 10% of the Arduino memory is obtained. 

## 4. Application of Proposed Network as a Cryptosystem

### 4.1. Chaos-Based Image Encryption Using Proposed 5-D Hyperjerk Oscillator Network 

Employing a permutation-substitution procedure, we propose the use of our 5-D hyperjerk chaos generator for image encryption, which requires refinements to our 5-D hyperjerk chaos generator as presented in (7):

(7) $$\left\{ \begin{array}{l} {\dot{x}}_{1}=x_{2}\mathrm{mod}1\\ {\dot{x}}_{2}=x_{3}\mathrm{mod}1\\ {\dot{x}}_{3}=x_{4}\mathrm{mod}1\\ {\dot{x}}_{4}=bx_{5}\mathrm{mod}1\\ {\dot{x}}_{5}=\left(-a_{0}x_{5}-a_{1}x_{3}-a_{2}x_{2}-a_{3}x_{1}-a_{4}x_{4}(x_{4}-l_{1})(x_{4}-l_{2})\right)\mathrm{mod}1 \end{array}\right.$$

**Figure 16:**
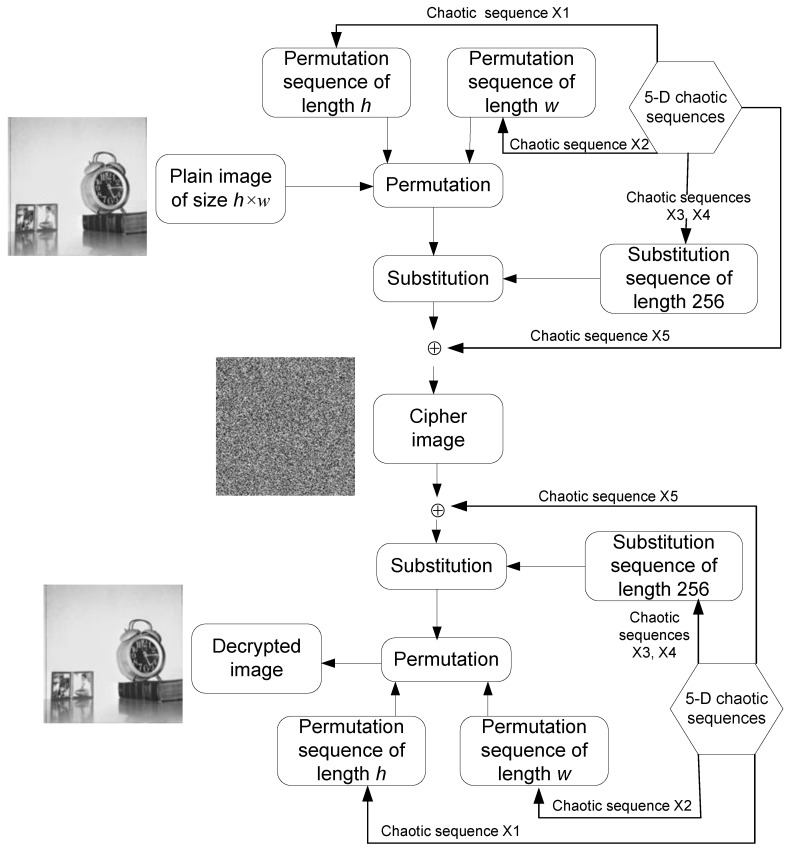
Outline of proposed permutation-substitution image encryption scheme based on sequences x1, x2, x3, x4, x5 of the 5-D hyperjerk chaotic generator.

This proposed scheme is outlined in Figure 16 and its execution is realized via the following steps where we use a plain image (P) and key parameters (*x*_1_, *x*_2_, *x*_3_, *x*_4_, *x*_5_, *a*_0_, *a*_1_, *a*_2_, *a*_3_, *a*_4_, *b*, *l*_1_, *l*_2_) for iterating 5-D hyperjerk chaos generator as input and the cipher-image (c) as output.

**Step 1**: Iterate the 5-D hyperjerk chaos generator for h*w times, where h*w is the size of the plain image *P,* which produces output is five sequences x1, x2, x3, x4, and x5 as output.
**Step 2:** Using the first sequence x1, construct a permutation sequence of length h with h distinct elements from 1 to h as follows:
  − Order the elements of first h elements and discard the first 10 elements in ascending order.
      Eh= order (x1(11:h+10))
  − Obtain the index of each element of the sequence Eh as a sequence x1(11:h+10).
      Ph=index (Eh in x1(11:h+10))
  **Step 3:** Using the first sequence x2, construct a permutation sequence of length w with distinct elements from 1 to w.
  − Order the elements of first w elements and discard the first 10 elements in ascending order.
      Ew= order (x2(11:w+10))
  − Obtain the index of each element of the sequence Ew as a sequence x2(11:h+10).
      Pw=index (Ew in x2(11:w+10))
**Step 4:** Using the first sequence the third and fourth sequences x3 and x4, construct the substitution sequence of length 256, which have 256 distinct elements in the range 0 to 255
  − Y=x3(11:266) + x4(11:266)
  − Order the elements of Y sequence in ascending order.
      Ey= order(Y)
  − Obtain the index of each element of the sequence Ey as a sequence Y.
      Sb=index (Ey in Y)
**Step 5:** Using the fifth sequence X5, construct the key matrix K with size h×w.
  $$K=fix\left(X5\times 10^{12}\right)\mathrm{mod}256$$
**Step 6:** Permute the plain image *P* using the permutation sequences Ph and Pw (which originate from Step 2 and Step 3, respectively), each targeting the rows and columns.
    for i=1 to h
     for j=1 to w
      Per(i,j)=P(Ph(i),Pw(j));
     end
    end
**Step 7:** Substitute the permutated image ‘Per’ (in Step 6) using Sb substitution sequence (in Step 4).
    Sub=zeros(a,b);
    for i=1 to h
     for j=1 to w
      Sub (i,j)=Sb(Per(i,j)+1);
     end
    end
**Step 8:** Perform bitwise XOR operation on substituted image ‘Sub’ (in Step 7) using key matrix K (in Step 5). C=bitxor(Sub,K)

### 4.2. Performance Tests

**Figure 17:**
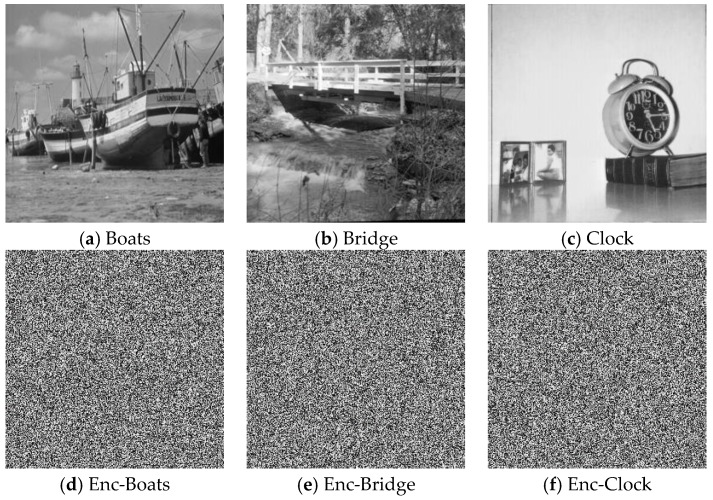
Visual test results showing the plain image and its cipher version.

To test the performances of our encryption scheme, we set system parameters and initial values within the window of coexisting attractors as $(a_{0},a_{1},a_{2},a_{3},a_{4},b,I_{1},I_{2})=$(1.5,3,3.454,1,1,3,1,2.6) respectively $\left(x_{1},x_{2},x_{3},x_{4},x_{5})=(0.7752,0.6733,0.9534,0.8735,0.8736\right)$. Further, we simulated implementation of the proposed scheme using 256×256 sized versions of the Boats, Bridge, and Clock greyscale images in Figure 17a–c on an Intel^®^ core^TM^ i5-2450M and 6 GB RAM workstation with a preinstalled MATLAB R2016b software. As seen from the outcome in Figure 17d–f, the encrypted images are visually imperceptible. However, the simple visual inspection remains insufficient to judge the quality of a good encryption scheme. It is well known that many encryption schemes have been successfully violated using simple statistical and differential analysis are widely used to successfully validate the efficiency of encryption schemes [42,43,44,45,46,47,48,49,50,51,52,53,54]. The robustness of our proposed technique is similarly established via these simple, yet important tests as presented in the remainder of this subsection.

#### 4.2.1. Statistical Tests

##### Correlation of Adjacent Pixels

In sensitivity analysis of encryption keys, quantitative analyses are undertaken using the correlation coefficient [40] metric. In such analysis, the neighbouring pixels of a plain image should be highly correlated with correlation coefficient close to unity (i.e., 1) in each direction. Furthermore, an ideal encryption scheme must produce cipher image with no correlation between neighboring pixels (i.e., correlation coefficient should be close to 0 in each direction). For this purpose, correlation coefficient is computed using the definition in (8).

(8) $$r_{xy}=\frac{\sum_{i=1}^{M}\left(x_{i}-\bar{x})(y_{i}-\bar{y}\right)}{\sqrt{\sum_{i=1}^{M}{\left(x_{i}-\bar{x}\right)}^{2}\sum_{i=1}^{M}{\left(y_{i}-\bar{y}\right)}^{2}}}$$

where a pixel *r* is defined by *r*(*x*_i_,*y*_i_) and *M* is the total number of pixels in the cipher image. Table 4 provides the correlation coefficients for the plain and encrypted versions of images in Figure 17 and from this table it is apparent that the input and encrypted images are highly correlated since correlation coefficient of the encrypted images are very close to 0 in each direction. Consequently, we conclude that the proposed encryption algorithm produces efficiently correlated ciphered images.

##### Histogram Tests

**Figure 18:**
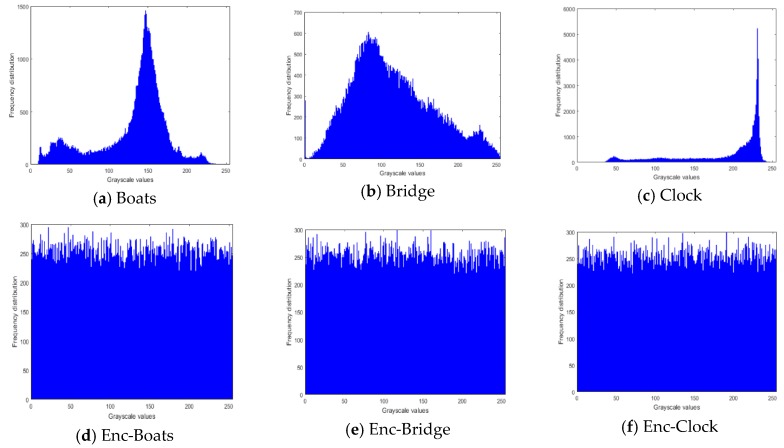
Histograms of original and encrypted images in Figure 17.

An image histogram is the representation of each pixel in the image with respect to its intensity value [55,56,57]. This analysis is very useful in deciding the statistical strength of an encryption algorithm. As a representation of incomprehensible information, the histogram of a cipher image is uniformly distributed, while the non-uniform nature of a pristine un-enciphered image depicts the details therein. Figure 18 presents the histograms of the plain and ciphered images used in our experiment, outcomes of which further establish the performance of our proposed scheme in resisting statistical manipulations to the content of the encrypted image.

##### Information Entropy

Another statistical metric that is widely used to assess the capability of a cipher scheme to resist statistical attacks is the measure of its information entropy. The distribution (entropy) of each pixel *x*_i_ with the probability *p*(*x*_i_) in a given image can be defined as:

(9) $$E(X)=-\sum_{i=1}^{2^{L}-1}p(x_{i})\log_{2}(p(x_{i}))$$

Given that a greyscale image has 256 possible values, the ideal entropy value should be close to 8. Table 5 provides information entropy values for the encrypted images in comparison with values obtained via previous studies as indicated in the table. 

#### 4.2.2. Differential Test: NPCR and UACI

In addition to performing well in the statistical tests reported above, a well design encryption algorithm should be very sensitive to slight changes in the composition of the plain image [51,52,53,54]. This sensitivity can be evaluated by computing the Number of Pixels Change Rate (NPCR) and the Unified Average Changing Intensity (UACI) which are defined in (10) and (11) respectively.

(10) $$NPCR=\frac{\sum_{i;j}D(i,j)}{w\times h}\times 100\%\;,D(i,j)=\{\begin{array}{c} 0\;\;if\;\;IC_{1}(i,j)=IC_{2}(i,j)\\ 1\;\;if\;\;IC_{1}(i,j)\neq IC_{2}(i,j) \end{array}$$

(11) $$UACI=\frac{100}{w\times h}\sum_{1}^{w}\sum_{1}^{h}\frac{\left|IC_{1}(i,j)-IC_{2}(i,j)\right|}{255}$$

where $IC_{1}$ and $IC_{2}$ are two encrypted images obtained from plain images different in just one pixel, *w* and *h* are the dimensions of the images. For an image to be uniformly distributed, the minimum expected values of NPCR and UACI should satisfy (12) and (13) respectively.

(12) $$NPCR_{\max}=(1-2^{8})\times 100=99.609375\%$$

(13) $$UACI_{\max}=\frac{\sum_{j=1}^{2^{8}-1}j(j+1)}{2^{8}(2^{8}-1)}\times 100=33.46354\%$$

The results in Table 6 validate the sensitivity and ability of images obtained via proposed scheme to withstand differential attacks aimed at violating their integrity. 

#### 4.2.3. Key Sensitivity Test

**Figure 19:**
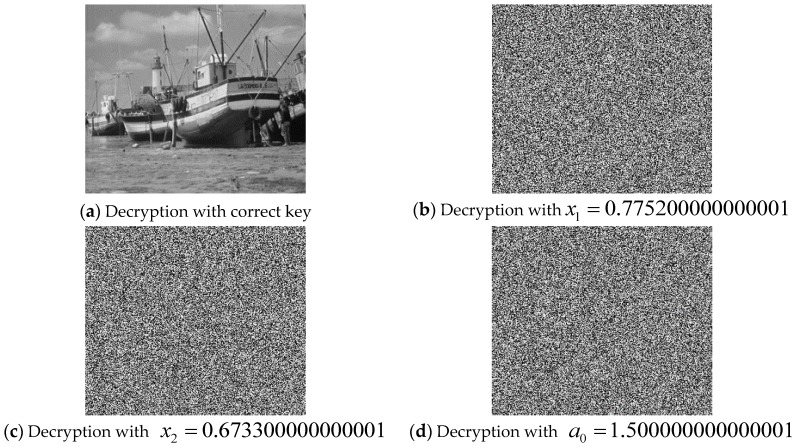
Correct decoding of Boats image and decoding by a slightly changed keys.

An efficient and robust encryption algorithm must show sensitivity to even the slightest changes in the composition of its secret key [38,39,40,41,55,56,57]. This is especially important in resisting brute force attacks. To evaluate the key sensitivity of our proposed scheme, the encrypted image is decrypted using four slightly different test keys. The results presented in Figure 19 show the impact of slight modifications to key parameters in yielding erroneous outcomes, i.e., ensuring the encrypted image is inaccessible unless with the exact key parameters.

#### 4.2.4. Time and Complexity Analysis

The speed of an algorithm depends on some important factors such as the specifications and structure of the CPU, the size of memory, the size of image, the software used, etc. To assess our algorithm with those in [43,52,53,54], we ensured a level playing ground by first attuning it with those in compared studies using 512 × 512 sized images. Second, we simulate the execution under the same environment: A laptop with Intel core^TM^ i5-2450M 6 GB RAM and a preinstalled MATLAB R2016b software. The total encryption time of the proposed approach includes iterating the 5-D hyperjerk chaos generation, constructing permutation sequences (Ph and Pw), constructing substitution sequence (Sb), and encryption process. Table 7 gives the detailed time analysis for each process in the encryption procedure. Consequently, only temporal constraints arising from diffusion and confusion procedures of each algorithm are assessed. Further, Table 8 provides the time analysis for our scheme based on the specs outlined in comparison with results from similar techniques as reported.

In addition to encryption time tests, we also undertook a complexity analysis [56] as outlined here. For uniformity and level playing ground, this analysis is done in terms of CPU operations required to execute the different methods. Therefore, each step of our proposed method as well as those to be used in the comparison are used to estimate the complexity cost.

- Step 1: (5*h*w) steps are required to iterate the chaotic map
- Step 2: (h^2^) steps each are required to retrieve *h* elements and obtain the index
- Step 3: (w^2^) steps each are required to retrieve *w* elements and obtain the index
- Step 4: (256*256) steps each are required to retrieve 256 elements, and obtain the index of *h* elements
- Step 5: (h*w) steps are each required for the multiplication mod operations
- Step 6: (h*w) steps are required for the permutation operation
- Step 7: (h*w) steps are required for substitution operation
- Step 8: (h*w) steps are required for number of exclusive-XOR operations in the final step

Therefore, the complexity of the algorithm proposed to execute the encryption procedure is *O*(*max*(h^2^, w^2^, h*w) which is an improvement over the complexity reported in [55].

#### 4.2.5. NIST Test

To establish the effectiveness of the presented encryption mechanism, we assessed the randomness property of the resulting fifth sequence (for example) as stipulated via NIST SP 800-22 tests, which are considered as the industry standard. These tests consist of 15 examinations that are performed on the fifth generated sequence with 106 bits length and as presented in Table 9 the generated sequence passed tests administered.

#### 4.2.6. Key Space Analysis 

A well-designed image encryption approach should have a sufficiently large key-space which is known as the several keys that can be used in brute-force attacks [38,39,40,41,55,56,57]. For our proposed technique, the image encryption algorithm utilizes the key parameters (*a*,*b*,*y*,*x*_1_,*x*_2_,*x*_3_,*x*_4_,*x*_5_) to generate the encryption key *K*. For context, suppose that the calculation precision (floating point operations) for each key is 10^16^, then the total key space of whole system is 10^128^, which is within thresholds expected from state-of-the-art encryption algorithms.

#### 4.2.7. Impact of Noise on the Transmission of Cipher Images

**Figure 20:**
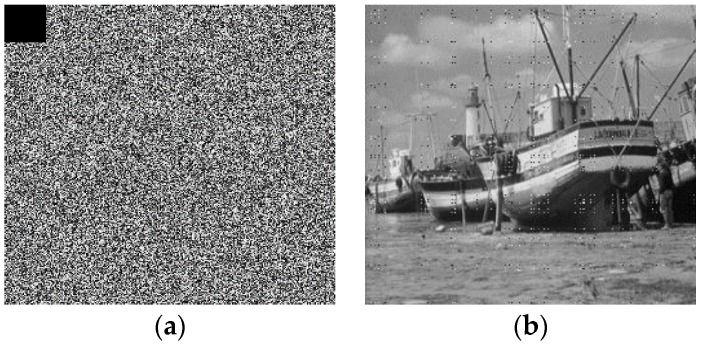
Effect of noise on the cipher Boats image: (**a**) Noise infected cipher image; (**b**) successful decrypted image.

In providing a thorough assessment of our proposed technique, it is important to evaluate the effect of noise on the cipher image during the transmission. For this, we consider a black cut out that is obtained by modifying 1024 pixels (32×32) in the encrypted image and setting their values to zero. We then execute the decryption procedure on the noisy encrypted image as presented in Figure 20. From this result we observe that despite the noise, the original boats image can be recovered with high visual fidelity (see Figure 20b). Consequently, we can infer the utility of our proposed scheme for public transmission.

## 5. Concluding Remarks 

This study has proposed, designed, and implemented a 5-D hyperjerk oscillator for chaos generation. Extensive numerical analysis employed showed that the proposed oscillator network comprised of diodes and resistors can produce nonlinearities (both square and cubic) required to generate chaos. Moreover, in addition to being cheaper than standard analogue multipliers, our proposed network was shown to exhibit the capacity to experience coexistence of hidden attractors in the phase space. Furthermore, we exploited the efficient cost-effective design of our network to explore deployment of its lightweight version as an image cryptosystem. Based on this application and outcomes of standard security analysis that were undertaken, the performance and utility of our proposed image encryption scheme were validated. Additionally, an Arduino UNO set up was utilized to implement the network and experimental results showed that our proposed chaos generator could have useful applications in emerging paradigms for information and communication security. For future and ongoing work, our study is being improved in the following directions. First, we note that despite their relative objectivity, statistical tests do not cover all aspects of cryptanalytical attacks. Therefore, following necessary refinements, we plan on integrating differential trails over the encryption process. These are reputed to be more powerful then permutation-only and substitution paradigms. Additionally, in ongoing work, we are exploring the use of Cobweb diagrams (representation of phase plot for digital systems) for chaos generation based on the Arduino platform. Insights from this and other improvements to this study will be used to improve image complexity analysis, develop faster and more robust encryption strategies for colour images aimed primarily at securing medical images for applications in telemedicine.

## Figures and Tables

**Table 1:** Strategies and parameter settings used to obtain coexisting bifurcations (as indicated in the table).

Figure Number	Graph Colour	Parameter Range	Sweeping Direction	Initial State (x1(0),x2(0),x3(0),x4(0),x5(0))
7	Green	$2\leq a_{2}\leq 4$	Downward	(4,0,0,0,0,0)
	Red	$2\leq a_{2}\leq 4$	Downward	(4.4,0,0,0,0,0)
	Blue	$3.212\leq a_{2}\leq 4$	Upward	(5.2,0,0,0,0,0)
	Black	$2\leq a_{2}\leq 4$	Downward	(0.4,0,0,0,0,0)
10	Green	$3.405\leq a_{2}\leq 3.454$	Downward	(4,0,0,0,0,0)
	Red	$3.405\leq a_{2}\leq 3.454$	Downward	(4.4,0,0,0,0,0)
	Blue	$3.405\leq a_{2}\leq 3.454$	Upward	(5.2,0,0,0,0,0)
	Black	$3.405\leq a_{2}\leq 3.454$	Downward	(0.4,0,0,0,0,0)
	Cyan	$3.405\leq a_{2}\leq 3.454$	Downward	(1.2,0,0,0,0)

**Table 2:** Numerical initial conditions for multistability analysis for selected parameters ${(a}_{0}\;,\;a_{1},\;a_{3}\;,\;a_{4}\;,\;b\;,\;l_{1}\;,\;l_{2})=(1.5\;,\;3\;,\;1\;,1\;,\;3\;,\;1\;,\;2.6)$.

Figure Number	Type of Coexistence	Control Parameter (a_2_)	Numerical Initial Conditions
8	One cycle and a chaotic attractor with fixed point	2.9	(4,0,0,0,0,0), (6,0,0,0,0,0)
11	Three different limit cycles and a chaotic attractor with fixed point	3.454	(a) (0.4,0,0,0,0), (1.2,0,0,0,0); (b) (5.2,0,0,0,0); (c) (4,0,0,0,0); (d) (4.4,0,0,0,0)

**Table 3:** Component values used in circuit simulation analysis.

Components	Property	Rating
*R*	Resistance	10 kΩ
*R_a_*_0_	Resistance	6.66 kΩ
*R_a_*_1_	Resistance	3.33 kΩ
*R_a_*_2_	Resistance	3.5 kΩ
*R_a_*_3_	Resistance	10 kΩ
*R_b_*	Resistance	3.33 kΩ
*R*_1_	Resistance	3.85 kΩ
*R*_2_	Resistance	277.77 kΩ
*R*_3_	Resistance	0.1 kΩ
***C*_i_(*i* = 1,…5)**	Capacitance	10ηF
***U*_i_(*i* = 1,…5)**	Operational Amplifier	TL084

**Table 4:** Correlation coefficients for the plain image and the related encrypted version.

	Correlation Coefficients					
Image	Plain Image			Cipher Version		
Direction	Diagonal	Horizontal	Vertical	Diagonal	Horizontal	Vertical
[10]	0.9466	0.9839	0.9526	−0.0474	−0.033	0.0068
[11]	0.9116	0.9282	0.9644	−0.0319	0.0245	0.0295
[55]	0.8888	0.9567	0.9239	−0.00012	0.0006	−0.0052
Proposed method						
Boats	0.9452	0.9266	0.8855	−0.0007	0.0007	−0.0015
Bridge	0.9203	0.9403	0.8866	−0.0027	0.0008	−0.001
Clock	0.9767	0.9578	0.9426	−0.0001	0.0007	−0.0023

**Table 5:** Assessment of information entropy for encrypted image.

Encryption Algorithm	Entropy
[10] Greyscale flower image	7.9969
[11] Cameraman image	7.9455
Proposed method	
Boats	7.9976
Bridge	7.9974
Clock	7.9975

**Table 6:** Comparative analysis of UACI and NPCR values with respect to encrypted image.

Encryption Algorithm	NPCR (%)	UACI (%)
[10] Grey flower image	99.15	33.21
[11] Cameraman image	99.34	33.61
	Proposed method	
Boats	99.62	33.69
Bridge	99.60	33.24
Clock	99.64	35.26

**Table 7:** Encryption time (in seconds).

Process	Image Size					
	32 × 32	64 × 64	128 × 128	256 × 256	512 × 512	1024 × 1024
Iterations for 5DHO chaos generation	0.000166	0.000728	0.002900	0.010600	0.041200	0.178700
Constructing permutation sequences (Ph and Pw)	0.000105	0.000172	0.000433	0.002200	0.009500	0.032100
Constructing substitution sequence (Sb)	0.000730	0.000730	0.000730	0.000730	0.000730	0.000730
Encryption process	0.001100	0.004500	0.014900	0.053700	0.062700	0.968700
Total time	0.002100	0.006130	0.018963	0.067230	0.114130	1.180230

**Table 8:** Encryption time (in seconds) where N.R. = Not reported implies that the parameter was not reported in the cited study.

Encryption Algorithm	Image Size					
	32 × 32	64 × 64	128 × 128	256 × 256	512 × 512	1024 × 1024
[43]	N.R	0.0045	0.0163	0.0629	0.2673	1.2157
[52]	N.R	N.R	N.R	0.0460	0.2300	0.9530
[53]	N.R	N.R	N.R	0.0790	0.2454	N.R
[54]	N.R	N.R	N.R	N.R	0.2141	N.R
Proposed method	0.0021	0.0062	0.0190	0.0672	0.1141	1.1802

**Table 9:** NIST SP 800-22 tests results.

Test-Name	P-Value	Result
Frequency	0.890240	Passed
Block-frequency	0.563092	Passed
DFT	0.378341	Passed
Rank	0.236565	Passed
Runs	0.089504	Passed
Longest runs of ones	0.172795	Passed
Overlapping templates	0.320178	Passed
No overlapping templates	0.465065	Passed
Universal	0.518372	Passed
Approximate entropy	0.844091	Passed
Linear complexity	0.042035	Passed
Cumulative sums (forward)	0.793995	Passed
Cumulative sums (reverse)	0.899532	Passed
Serial test 1	0.179396	Passed
Serial test 2	0.662233	Passed
Random excursions x = 1	0.207249	Passed
Random excursions variant x = 1	0.042985	Passed
